# A comparison of estimated drug costs of potentially inappropriate medications between older patients receiving nurse home visit services and patients receiving pharmacist home visit services: a cross-sectional and propensity score analysis

**DOI:** 10.1186/s12913-015-0732-4

**Published:** 2015-02-21

**Authors:** Jun Hamano, Sachiko Ozone, Yasuharu Tokuda

**Affiliations:** 1 Division of Clinical Medicine, Faculty of Medicine, University of Tsukuba, Tsukuba, Japan; 2 Primary Care and Medical Education, Graduate School of Comprehensive Human Sciences, University of Tsukuba, Tsukuba, Japan; 3 Japan Community Healthcare Organization, 3-22-12 Takanawa, Tokyo, Japan

## Abstract

**Background:**

There have been no multicenter studies that estimated the relations of either nurse or pharmacist home visit program to drug costs of potentially inappropriate medications (PIMs). This study aimed to establish whether patients who used nurse or pharmacist home visit programs (nurse or pharmacist program) had lower drug costs of PIMs than those who did not use nurse or pharmacist programs for older patients living at home.

**Methods:**

This cross-sectional study was conducted in home care settings in Japan, involving 430 patients aged 65 or older, of whom 276 were female. All received regular home visits from five clinics between May and December 2013. After the PIMs were identified with the Screening Tool of Older Persons' potentially inappropriate Prescriptions (STOPP) criteria, we estimated the drug costs based on actual pharmaceutical prices and measured against who using nurse or pharmacist programs after a propensity score weighted adjustment.

**Results:**

Patients who used nurse programs had lower drug cost of PIMs than those who did not use, but it was not significantly different (5.9 ± 13.1 vs 7.1 ± 13.9 USD per month, P = 0.199). The cost of PIMs for patients who used pharmacist programs also had no difference. (7.2 ± 14.5 vs 5.5 ± 11.5 USD per month, P = 0.06). In the patient groups who used nurse programs, patients who also used pharmacist programs had significantly higher costs of PIMs than those who used only nurse programs (5.5 ± 13.9 vs 2.5 ± 6.0 USD per month, P = 0.006). In patients group who did not use pharmacist programs, patients who only used nurse programs had significantly lower costs of PIMs than those who did not use nurse programs (3.6 ± 7.7 vs 5.8 ± 12.7 USD per month, P = 0.022).

**Conclusions:**

Patients who used nurse program have a trend towards lower drug costs of PIMs than those who used nurse and pharmacist program or pharmacist program alone. Although this study tried to adjust the potential confounders as possible as we could by using propensity score analysis, further studies are needed to confirm our results.

## Background

Prescription medications are an essential component of the care of older patients. Although some medications can cure and prevent disease, inappropriate medication may have detrimental effects. Potentially inappropriate medications (PIMs) have been defined as medications that carry more risks than benefits, those with clinically significant drug–drug or drug–disease interactions [1,2].

Several studies have shown that PIMs among older patients are correlated with increased adverse drug reactions (ADRs) [3-6], health care utilization [7-9], death [10,11], poor adherence [12,13] and greater economic burden [7,14,15]. The cost of potentially inappropriate medications is estimated to be high [16-18], and this has become an important public health issue worldwide [2]. PIMs among older home care patients are common [19], and an important issue in developed countries with an aging population, such as Japan, where the number of older home care patients is predicted to increase rapidly in the future.

Several tools are used to detect PIMs in older people [20], such as the Beers’ criteria [21], the Screening Tool of Older Persons' potentially inappropriate Prescriptions (STOPP) [22,23] and the Improved Prescribing in the Elderly Tool (IPET) [24]. Recent studies showed that the STOPP criteria have high sensitivity for detecting potential drug-related problems [25] and are more sensitive in detecting PIMs than the Beers’ criteria [26].

Several intervention studies have revealed that a multidisciplinary approach for older patients could reduce the number of patients with PIMs [27,28] and detect a high proportion of clinically relevant DRPs [29,30]. One previous study conducted in home care settings revealed that a medication review by a community pharmacist could reduce the number of medications, but it was not clear about the relations of pharmacist intervention to drug costs of PIMs [31]. As far as we know, there has been no multicenter study that estimated the relations of either nurse or pharmacist home visit program to drug cost of PIMs identified by the STOPP criteria for older patients in home care settings. This study aimed to validate whether the patients who using either nurse or pharmacist home visit programs had lower drug costs of potentially inappropriate medication based on actual pharmaceutical prices than who not using nurse or pharmacist home visit programs for older patients in a home care setting.

## Methods

This cross-sectional study was conducted at five clinics in Japan between May and December 2013. These clinics provide primary care by ambulatory service and home visit services for community residents, and each clinics collaborate with home visit nurses and pharmacists depending on the situation. None of these doctors were familiar with the STOPP criteria at the starting time of study. In general, nurse home visit programs in Japan provide hands-on care at home, for example to help bathing, to promote physical activity and to coordinate living environment. Pharmacist home visit programs in Japan usually consult with patient regarding expected or unexpected effects of drugs and monitor the adherence of prescription at home. The frequency of their home visit depended on patients’ conditions and needs for care, typically twice or four times a month by nurse and a once or twice a month by pharmacist.

Although, the primary care doctors responsible for care of individual patients typically recommend using the home visit program and patients or their family could decide to use or not, the patients and their family could use the programs whenever they requested.

The study was approved by the ethics committee of the Mito Kyodo General Hospital and was conducted according to the principles expressed in the Declaration of Helsinki. We included all patients who were 65 years or older and who satisfied our inclusion criteria, which were:

1. patients received home visit services regularly by their doctors at least twice a month for over a month, and
2. patients had been regularly prescribed medications by the clinic, excluding topical drugs.

We used medical records to collect patients' background information, which included age, sex, estimated glomerular filtration rate (eGFR: ml/min), serum albumin (mg/dl), availability of overview of ambulation and drug use, underlying medical conditions, whether they lived with or without family, and whether they used a home visit nurse or pharmacist.

We had collected the copies of prescription contents by single monthly basis during the survey period which was sent from each clinic and confirmed the medication.

We defined PIMs as having occurred when at least one of the original STOPP criteria was met (Table 1). We calculated the total monthly drug cost of all patients and the monthly drug cost of PIMs. We estimated the drug cost based on actual pharmaceutical prices listed by the regulatory committee at the Ministry of Health, Labour and Welfare, Japanese government.

**Table 1:** **STOPP screening criteria** [22]

	STOPP criteria
A. Cardiovascular system	
1	Digoxin at a long-term dose >125 mg/d with impaired renal function
2	Loop diuretic as first-line monotherapy for hypertension or for dependent ankle edema only
3	Thiazide diuretic with a history of gout
4	Noncardioselective b-blocker with chronic obstructive pulmonary disease
5	b-blocker in combination with verapamil
6	Use of diltiazem or verapamil with New York Heart Association class 3 or 4 heart failure
7	Calcium-channel blockers with chronic constipation
8	Use of aspirin and warfarin in combination without histamine H2 receptor antagonist
	(except cimetidine) or proton pump inhibitor
9	Dipyridamole as monotherapy for cardiovascular secondary prevention
10	Aspirin with no history of coronary, cerebral or peripheral vascular symptoms
	or occlusive arterial event or with a past history of peptic ulcer disease
	without histamine H2 receptor antagonist or proton pumpinhibitor or at dose >150 mg/d
	or to treat dizziness not clearly attributed to cerebrovascular disease
11	Warfarin for first, uncomplicated deep venous thrombosis for longer than 6-month duration
	or for first, uncomplicated pulmonary embolus for longer than 12-month duration
12	Aspirin, clopidogrel, dipyridamole, or warfarin with concurrent bleeding disorder
B. CNS and psychotropic drugs	
1	Tricyclic antidepressants with dementia or with glaucoma or with cardiac conductive abnormalities
	or with constipation or with prostatism or prior history of urinary retention or with an opiate
	or calcium-channel blocker
2	Long-term (>3 month) use of long-acting benzodiazepines, and with long-acting metabolites
3	Long-term (>1 month) neuroleptics as long-term hypnotics or in those with parkinsonism
4	Phenothiazines in patients with epilepsy
5	Anticholinergics to treat extrapyramidal side effects of neuroleptic medications
6	Selective serotonin reuptake inhibitors with a history of clinically significant hyponatremia
	(noniatrogenic hyponatremia <130 mmol/L within the previous 2 months)
7	Prolonged use (>1 week) of first-generation antihistamines
C. Gastrointestinal system	
1	Diphenoxylate, loperamide or codeine phosphate for treatment of diarrhea of unknown cause
	or severe infective gastroenteritis
2	Prochlorperazine or metoclopramide with parkinsonism
3	Proton pump inhibitors for peptic ulcer disease at full therapeutic dosage for >8 weeks
4	Anticholinergic antispasmodic drugs with chronic constipation
D. Respiratory system	
1	Theophylline as monotherapy for chronic obstructive pulmonary disease
2	Systemic corticosteroids instead of inhaled corticosteroids for maintenance therapy
	in moderate-to-severe chronic obstructive pulmonary disease
3	Nebulized ipratropium with glaucoma
E. Musculoskeletal system	
1	NSAID with history of peptic ulcer disease or gastrointestinal bleeding, unless with concurrent
	histamine H2 receptor antagonist, proton pump inhibitor, or misoprostol
2	NSAID with moderate-to-severe hypertension or with heart failure
3	Long-term use of NSAID (>3 months) for relief of mild joint pain in osteoarthritis
4	Warfarin and NSAID together
5	NSAID with chronic renal failure
6	Long-term corticosteroids (>3 months) as monotherapy for rheumatoid arthritis
	or osteoarthritis
7	Long-term NSAID or colchicine for chronic treatment of gout where there is
	no contraindication to allopurinol
F. Urogenital system	
1	Bladder antimuscarinic drugs with dementia or with chronic glaucoma
	or with chronic constipation or with chronic prostatism
2	a-blockers in males with frequent incontinence
3	a-blockers with long-term urinary catheter in situ (>2 months)
G. Endocrine system	
1	Glibenclamide or chlorpropamide with type 2 diabetes mellitus
2	b-blockers in those with diabetes mellitus and frequent hypoglycemic episodes
3	Estrogens with a history of breast cancer or venous thromboembolism
4	Estrogens without progestogen in patients with intact uterus
H. Drugs that adversely affect those prone to falls (at least 1 fall in past 3 months)	
1	Benzodiazepines
2	Neuroleptic drugs
3	First-generation antihistamines
4	Vasodilator drugs known to cause hypotension in those with persistent postural hypotension
5	Long-term opiates in those with recurrent falls
I. Analgesic drugs	
1	Use of long-term powerful opiates as first-line therapy for mild-to-moderate pain
2	Regular opiates for more than 2 weeks in those with chronic constipation
	without concurrent use of laxatives
3	Long-term opiates in those with dementia unless indicated for palliative care
	or management of moderate-to-severe chronic pain syndrome
J. Duplicate drug classes	
1	Any regular duplicate drug class prescription, such as two concurrent opiates, NSAIDs,
	serotonin-specific reuptake inhibitors, loop diuretics, and ACE inhibitors

### Statistical methods

We used Student’s *t* test for continuous variables and Pearson’s *χ*^2^ test or Fisher’s exact test for categorical variables to test for significant associations between patients’ baseline characteristics and the use of the home visit nurse or pharmacist. We used a propensity score weighting technique to assess the association between the monthly drug cost of PIMs and the use of the home visit nurse or pharmacist and to adjust for confounding factors.

For the propensity score analysis, we selected the variables as those that 1) were hypothesized to be strongly associated with the use of the home visit nurse or pharmacist and PIMs, and 2) were hypothesized to be associated with PIMs, and excluded those that 1) were associated with other aspects as well as the use of the home visit nurse and pharmacist, 2) were affected by the use of the home visit nurse or pharmacist, 3) perfectly predict the use of the home visit nurse and pharmacist [32].

We then used logistic regression to calculate the propensity score, categorizing the patient age into three groups: 65–74, 75–84, and >85 years.

The logistic regression model for the propensity score for the use of the nurse home visit programs (PS for nurse) included age category, sex, availability of overview on ambulation and drug use, whether they lived with or without family, the use of the home visit pharmacist, number of prescriptions and underlying medical conditions (constipation, hypertension, dementia, cerebral infarction/transient ischemic attack, diabetes mellitus, atrial fibrillation, progressive malignancy, hyperuricemia/gout, heart failure, dyslipidemia, Parkinson’s Disease/Parkinson’s syndrome, osteoporosis, chronic obstructive pulmonary disease, peripheral artery occlusive disease, cerebral/subarachnoid hemorrhage, osteoarthritis) as variables.

We also developed the propensity score for the use of the pharmacist home visit programs (PS for pharmacist) using a logistic regression model that included the same variables as for the nurse home visit programs, except that use of the pharmacist home visit programs was changed to use of the nurse home visit program.

To examine the association between the monthly drug cost of PIMs and use of the nurse or pharmacist home visit programs, we used a multivariate logistic regression analysis and included use of the home visit programs with the propensity score as variables. All analyses were conducted using SPSS-J (ver. 22.0; IBM, Tokyo, Japan).

## Results

### Demography

We included 430 patients in this study, of whom 276 were female. Table 2 shows detailed patient background information. The mean patient age was 85.0 ± 8.3 years. The main underlying medical conditions were constipation in 243 patients (56.5%), hypertension in 228 patients (53.0%), and dementia in 218 patients (50.7%). Nearly half, 203 patients (47.2%), had used the nurse home visit programs and 182 (42.3%) had used the pharmacist home visit programs. Almost one fifth, 78 patients (18.1%), had used both nurse and pharmacist home visit programs (Table 2). Over one quarter, 123 patients (28.6%), had not used either program. Propensity score weighting successfully balanced the observed differences in patient background and the use of the two home visit programs (Table 2).

**Table 2:** **Patient background (n = 430)**

		Before propensity score weighted adjustment						After propensity score weighted adjustment					
	All patients	Using nurse home visit programs			Using pharmacist home visit programs			Using nurse home visit programs (weighted by PS for nurse)			Using pharmacist home visit programs (weighted by PS for pharmacist)		
	(n = 430);n (%)	Patients who used nurse home visit programs (n = 203); n (%)	Patients who did not used nurse home visit programs (n = 227); n (%)	P-value	Patients who used pharmacist home visit programs (n = 182); n (%)	Patients who did not used pharmacist home visit programs (n = 248); n (%)	P-value	Patients who used nurse home visit programs (%)	Patients who did not used nurse home visit programs (%)	P-value	Patients who used pharmacist home visit programs (%)	Patients who did not used pharmacist home visit programs (%)	P-value
Gender				0.013			0.073			0.924			0.950
Male	154 (35.8%)	85 (41.9%)	69 (30.4%)		74 (40.7%)	80 (32.3%)		(34.3%)	(34.0%)		(35.6%)	(35.4%)	
Female	276 (64.2%)	118 (58.1%)	158 (69.6%)		108 (59.3%)	168 (67.7%)		(65.7%)	(66.0%)		(64.4%)	(64.6%)	
Mean age (years ± standard deviation)	85.0 ± 8.3	84.3 ± 8.6	85.7 ± 8.0	0.07	84.7 ± 8.1	85.2 ± 8.4	0.523	85.5 ± 8.5	84.9 ± 8.1	0.298	85.3 ± 8.3	85.0 ± 8.2	0.575
Age (years)				0.004			0.177			0.909			0.976
65-74	55 (12.8%)	29 (14.3%)	26 (11.5%)		22 (12.1%)	33 (13.3%)		(12.0%)	(12.1%)		(12.9%)	(12.4%)	
75-84	126 (29.3%)	73 (36.0%)	53 (23.3%)		62 (34.1%)	64 (25.8%)		(30.6%)	(31.9%)		(28.8%)	(29.0%)	
≥85	249 (57.9%)	101 (49.8%)	148 (65.2%)		98 (53.8%)	151 (60.9%)		(56.0%)	(56.0%)		(58.3%)	(58.5%)	
Independent ambulation	199 (46.3%)	71 (35.0%)	128 (56.4%)	<0.001	91 (50.0%)	108 (43.5%)	0.185	(45.3%)	(45.6%)	0.925	(45.9%)	(46.1%)	0.934
Independent drug overview	115 (26.7%)	44 (21.7%)	71 (31.3%)	0.025	54 (29.7%)	61 (24.6%)	0.24	(22.8%)	(24.8%)	0.482	(27.4%)	(26.7%)	0.812
Living with family	381 (88.6%)	190 (93.6%)	191 (84.1%)	0.002	155 (85.2%)	226 (91.1%)	0.054	(87.3%)	(88.4%)	0.626	(88.7%)	(88.1%)	0.774
Using home visit programs by nurse	203 (47.2%)	-	-	-	78 (42.9%)	125 (50.4%)	0.121	-	-	-	(47.7%)	(47.1%)	0.855
Using home visit programs by pharmacist	182 (42.3%)	78 (38.4%)	104 (45.8%)	0.121	-	-	-	(41.5%)	(42.2%)	0.844	-	-	-
Underlying medical conditions													
Constipation	243 (56.5%)	113 (55.7%)	130 (57.3%)	0.738	115 (63.2%)	128 (51.6%)	0.017	59.6%	59.4%	0.955	55.4%	56.2%	0.818
Hypertension	228 (53.0%)	84 (41.4%)	144 (63.4%)	<0.001	101 (55.5%)	127 (51.2%)	0.379	49.5%	51.8%	0.513	52.8%	53.2%	0.907
Dementia	218 (50.7%)	87 (42.9%)	131 (57.7%)	0.002	89 (48.9%)	129 (52.0%)	0.523	52.5%	54.2%	0.601	50.9%	51.5%	0.860
Cerebral infarction/transient ischemic attack	104 (24.2%)	45 (22.2%)	59 (26.0%)	0.355	44 (24.2%)	60 (24.2%)	0.997	26.8%	24.5%	0.456	24.7%	25.1%	0.906
Osteoporosis	95 (22.1%)	38 (18.7%)	57 (25.1%)	0.111	47 (25.8%)	48 (19.4%)	0.11	26.0%	22.9%	0.299	22.1%	22.5%	0.898
Diabetes mellitus	82 (19.1%)	33 (16.3%)	49 (21.6%)	0.16	43 (23.6%)	39 (15.7%)	0.039	20.2%	19.1%	0.691	19.2%	19.7%	0.852
Coronary artery disease	59 (13.7%)	27 (13.3%)	32 (14.1%)	0.811	28 (15.4%)	31 (12.5%)	0.39	13.3%	15.3%	0.410	11.8%	13.1%	0.544
Atrial fibrillation	55 (12.8)%	29 (14.3%)	26 (11.5%)	0.38	27 (14.8%)	28 (11.3%)	0.277	12.9%	12.3%	0.776	12.7%	12.2%	0.816
Dyslipidemia	51 (11.9%)	17 (8.4%)	34 (15.0%)	0.034	27 (14.8%)	24 (9.7%)	0.102	9.9%	11.8%	0.358	12.2%	12.6%	0.847
Benign prostatic hypertrophy	47 (10.9%)	24 (11.8%)	23 (10.1%)	0.575	22 (12.1%)	25 (10.1%)	0.51	10.1%	11.6%	0.485	9.9%	10.8%	0.685
Progressive malignancy	34 (7.9%)	23 (11.3%)	11 (4.8%)	0.013	14 (7.7%)	20 (8.1%)	0.888	7.5%	7.1%	0.807	7.2%	7.7%	0.751
Hyperuricemia/gout	32 (7.4%)	9 (4.4%)	23 (10.1%)	0.025	19 (10.4%)	13 (5.2%)	0.042	9.2%	7.8%	0.473	8.1%	8.2%	0.951
Heart failure	30 (7.0%)	17 (8.4%)	13 (5.7%)	0.282	14 (7.7%)	16 (6.5%)	0.618	5.0%	7.3%	0.163	6.2%	5.9%	0.815
Parkinson's Disease/Parkinson's syndrome	28 (6.5%)	14 (6.9%)	14 (6.2%)	0.76	13 (7.1%)	15 (6.0%)	0.649	7.5%	7.8%	0.866	6.2%	6.6%	0.840
Chronic obstructive pulmonary disease	27 (6.3%)	19 (9.4%)	8 (3.5%)	0.013	17 (9.3%)	10 (4.0%)	0.025	5.9%	3.8%	0.156	6.0%	5.2%	0.592
Peripheral artery occlusive disease	27 (6.3%)	7 (3.4%)	20 (8.8%)	0.022	16 (8.8%)	11 (4.4%)	0.066	4.0%	5.7%	0.253	6.0%	5.6%	0.816
Cerebral/subarachnoid hemorrhage	26 (6.0%)	11 (5.4%)	15 (6.6%)	0.606	17 (9.3%)	9 (3.6%)	0.014	5.2%	6.4%	0.452	6.0%	6.3%	0.846
Osteoarthritis	21 (4.9%)	6 (3.0%)	15 (6.6%)	0.079	9 (4.9%)	12 (4.8%)	0.96	5.6%	5.0%	0.658	5.1%	4.9%	0.919
Number of prescriptions (mean ± standard deviation)	6.1 ± 3.0	6.3 ± 3.0	5.8 ± 3.0	0.065	6.6 ± 3.1	5.6 ± 2.8	<0.001	6.2 ± 3.0	6.0 ± 3.1	0.427	5.9 ± 3.0	6.0 ± 3.0	0.879

### Specific prescriptions of PIMs

By the STOPP criteria, 34.0% of the study population received at least one PIM. We found that the most common prescriptions resulting in PIMs were (a) calcium-channel blockers in patients with chronic constipation (74 patients, 17.2%), (b) long-term use of NSAIDs for relief of mild joint pain in osteoarthritis (16 patients, 3.7%), (c) long-term use of long-acting benzodiazepines (15 patients, 3.5%), and (d) NSAIDs in patients with moderate-to-severe hypertension or with heart failure (14 patients, 3.3%) (Table 3).

**Table 3:** **Most frequent prescriptions resulting in PIMs**

	Prescription	(n = 430); n (%)
1	Calcium-channel blockers with chronic constipation	74 (17.2%)
2	Long-term use of NSAID (>3 months) for relief of mild joint pain in osteoarthritis	16 (3.7%)
3	Long-term (>1 month) use of long-acting benzodiazepines, and with long-acting metabolites	15 (3.5%)
4	NSAID with moderate-to-severe hypertension or with heart failure	14 (3.3%)
5	Loop diuretic as first-line monotherapy for hypertension or for dependent ankle edema only	13 (3.0%)
6	Any regular duplicate drug class prescription, such as two concurrent opiates, NSAIDs, serotonin-specific reuptake inhibitors, loop diuretics, and ACE inhibitors	11 (2.6%)
7	Benzodiazepines for patients who have had at least 1 fall in the past 3 months	10 (2.3%)

### The cost of PIMs after propensity score weighted adjustment

After propensity score weighted adjustment, we compared the total cost of PIMs per patient per month in USD (100 Japanese Yen = 1 USD) in all patients and each subgroup (Table 4, 5). Although it was not significantly different, patients who used the pharmacist home visit programs had higher drug costs of PIMs (7.2 ± 14.5 vs 5.5 ± 11.5 USD per month, P = 0.06) (Table 4). On the other hand, the drug cost of PIMs for patients who used the nurse home visit programs was lower than those who did not use, but it was not significantly different (5.9 ± 13.1 vs 7.1 ± 13.9 USD per month, P = 0.199) (Table 5). In the patient groups who used nurse home visiting programs, patients who also used pharmacist home visiting programs had significantly higher costs of PIMs than those who used only nurse home visit programs (5.5 ± 13.9 vs 2.5 ± 6.0 USD per month, P = 0.006) (Table 4). In the patient groups who did not use pharmacist home visit programs, patients who only used nurse home visit programs had significantly lower costs of PIMs than those who did not use nurse home visit programs (3.6 ± 7.7 vs 5.8 ± 12.7 USD per month, P = 0.022) (Table 5).

**Table 4:** **The cost of PIMs in patients group who used nurse home visit or not**

(Total cost of PIMs per patient per month in USD)						
		Using pharmacist home visit programs (weighted by PS for pharmacist)				
	n	Patients who used pharmacist home visit programs (n = 182)	Patients who did not use pharmacist home visit programs (n = 248)	Difference between two groups	95% CI	P-value
All patients^†^	430	7.2 ± 14.5	5.5 ± 11.5	−1.7	−3.5 - 0.07	0.06
Patients who used nurse home visit programs^†^	203	5.5 ± 13.9	2.5 ± 6.0	−2.9	−5.0 - -0.83	0.006
Patients who did not use nurse home visit programs^†^	227	8.7 ± 15.7	8.1 ± 14.2	−0.66	−3.4 - 2.1	0.64

^†^drug cost (USD) ± standard deviation.

**Table 5:** **The cost of PIMs in patients group who used pharmacist home visit or not**

(Total cost of PIMs per patient per month in USD)						
		Using nurse home visit programs (weighted by PS for nurse)				
	n	Patients who used nurse home visit programs (n = 203)	Patients who did not use nurse home visit programs (n = 227)	Difference between two groups	95% CI	P-value
All patients^†^	430	5.9 ± 13.1	7.1 ± 13.9	1.2	−0.63 - 3.0	0.199
Patients who used pharmacist home visit programs^†^	182	9.1 ± 17.8	8.8 ± 15.3	−0.22	−3.7 - 3.2	0.898
Patients who did not use pharmacist home visit programs^†^	248	3.6 ± 7.7	5.8 ± 12.7	2.2	0.31 - 4.0	0.022

^†^drug cost (USD) ± standard deviation.

## Discussion

As far as we know, this is the first study to estimate the relations of nurse and pharmacist home visit program to drug costs of PIMs identified by the STOPP criteria for older patients in home care settings.

The important finding of this study is that those who used nurse home visit program have a trend towards lower drug costs of PIMs than those who used nurse and pharmacist home visit program or pharmacist home visit program alone.

There are several explanations about this finding. First, pharmacist home visit programs tend to involve patients with complicated conditions and PIMs might have been actually appropriate in these circumstances.

Second, although pharmacists understood the concept and meaning of PIMs, pharmacist home visit programs may be more difficult to help for improving the prescription than nurse home visit programs. This hypothesis is based on the situation of pharmacist home visit programs in Japan. In general, pharmacist home visit programs in Japan are expected to encourage to keep the drug adherence and to identify the symptoms as possible drug side effects. On the other hand, pharmacists of home visit programs usually did not receive the details of patients’ medical history from doctors and were not expected to give advice to doctors about prescription, although nurse home visit program usually is provided about the details of patients’ medical history and often be required to give advice to their patients about prescriptions from doctors in Japan.

Third, there might have been other unidentified confounding variables that might affect the effectiveness of home visit programs to the drug costs of PIMs. We need to conduct further research to reveal factors, which may affect the drug costs of PIMs in home care setting prospectively.

This study had four main limitations. First, we might not be able to assess the all of potential confounders that might affect the drug cost of PIMs in home care setting and the effectiveness of home visit program.

Second, because of its cross-sectional nature, there might have been several potential confounders, we could assess in this study, which could affect the drug costs of PIMs and the effectiveness of home visit program. We performed our analysis to adjust the potential confounders as possible as we could by using propensity scores.

Third, our study sample may not be representative of older home care patients, because it was carried out at only a few institutions in Japan. Further work is needed to carry out a larger study with the greater number of institutions in Japan and other countries.

Finally, we cannot draw conclusions about the effectiveness of nurse and pharmacist home visit program to drug cost of PIMs, because our study was not an intervention study. We would need to carry out further research, including a longitudinal intervention study, to assess the effectiveness of nurse and pharmacist home visit program to drug cost of PIMs.

## Conclusion

In conclusion, those who used nurse home visit program have a trend towards lower drug costs of PIMs than those who used nurse and pharmacist home visit program or pharmacist home visit program alone. Although, this study aimed to adjust the potential confounders as possible as we could by using propensity score analysis, caution may be needed about interpretation of this study. Further research is needed to consider all of potential confounders associated with the drug costs of PIMs in home care setting and using home visit program.
